# Toll-like receptor-3 contributes to the development of aortic valve stenosis

**DOI:** 10.1007/s00395-023-00980-9

**Published:** 2023-02-01

**Authors:** Sven Thomas Niepmann, Nicola Willemsen, Ann Sophie Boucher, Marta Stei, Philip Goody, Andreas Zietzer, Marko Bulic, Hannah Billig, Alexandru Odainic, Christina Katharina Weisheit, Christine Quast, Matti Adam, Susanne V. Schmidt, Farhad Bakhtiary, Felix Jansen, Georg Nickenig, Eike Latz, Sebastian Zimmer

**Affiliations:** 1 Heart Center Bonn, Clinic for Internal Medicine II, University Hospital Bonn, Bonn, Germany; 2 Clinic for Cardiology, University Hospital Cologne, Cologne, Germany; 3 Cardiovascular Research Laboratory, Division of Cardiology, Pulmonary Diseases and Vascular Medicine, Medical Faculty, Heinrich-Heine University, Düsseldorf, Germany; 4 Institute of Innate Immunity, University Hospital Bonn, Bonn, Germany; 5 Heart Center Bonn, Clinic for Heard Surgery, University Hospital Bonn, Bonn, Germany; 6 Department of Anaesthesiology and Intensive Care Medicine, University Hospital Bonn, Bonn, Germany; 7 Department of Microbiology and Immunology, The Peter Doherty Institute for Infection and Immunity, University of Melbourne, Melbourne, VIC Australia

**Keywords:** Aortic valve stenosis, Toll-like receptors, Innate immune system, Valvular interstitial cells, Valvular endothelial cells

## Abstract

Aortic valve stenosis (AS) development is driven by distinct molecular and cellular mechanisms which include inflammatory pathways. Toll-like-receptor-3 (TLR3) is a lysosomal pattern-recognition receptor that binds double-stranded RNA and promotes pro-inflammatory cellular responses. In recent years, TLR3 has emerged as a major regulator of vascular inflammation. The exact role of TLR3 in the development of AS has not been investigated. Isolated human valvular interstitial cells (VICs) were stimulated with the TLR3-agonist polyIC and the resulting pro-inflammatory and pro-osteogenic response measured. Severe AS was induced in wildtype- and TLR3^−/−^ mice via mechanical injury of the aortic valve with a coronary springwire. TLR3 activation was achieved by polyIC injection every 24 h after wire injury, while TLR3 inhibition was realized using Compound 4a (C4a) every 48 h after surgery. Endothelial mesenchymal transition (EndoMT) of human valvular endothelial cells (VECs) was assessed after polyIC stimulation. Stimulation of human VICs with polyIC promoted a strong inflammatory and pro-osteogenic reaction. Similarly, injection of polyIC marginally increased AS development in mice after wire injury. AS induction was significantly decreased in TLR3^−/−^ mice, confirming the role of endogenous TLR3 ligands in AS pathology. Pharmacological inhibition of TLR3 with C4a not only prevented the upregulation of inflammatory cytokines and osteogenic markers in VICs, and EndoMT in VECs, but also significantly abolished the development of AS in vivo. Endogenous TLR3 activation significantly contributes to AS development in mice. Pharmacological inhibition of TLR3 with C4a prevented AS formation. Therefore, targeting TLR3 may be a viable treatment option.

**Supplementary Information:**

The online version contains supplementary material available at 10.1007/s00395-023-00980-9.

## Introduction

Aortic valve stenosis (AS) is the most common valvular disease requiring therapeutic intervention in the western world [12, 21]. AS is a slowly progressive disease and most patients remain asymptomatic for a long time. Once patients develop symptoms, the mortality rate increases to 50% over 2 years [18, 23]. Therefore, current guidelines recommend valve replacement in symptomatic patients with severe AS. So far, no medical therapies have been developed to slow disease progression or to positively influence patient outcome. Surgical or interventional valve replacement remains the only option to treat aortic valve stenosis [34].

The lack of treatment alternatives reflects our poor understanding of the pathomechanisms driving AS development. Traditionally, AS has been considered a passive, degenerative consequence of many years of mechanical stress on the valve. However, recent experimental evidence has disputed this notion and the current understanding of AS finds inflammatory processes to be responsible for the development and progression of AS [10, 26]. It has been shown that large populations of inflammatory cells, such as neutrophils, B cells, and mast-cells, can be found in stenotic human aortic valves [6, 22, 36]. In fact, the known stages of AS—endothelial dysfunction, lipid deposition, extra-cellular matrix remodeling, and calcification—are in large parts orchestrated by inflammation, which is mainly characterized by activation of the innate immune system [7, 24].

The innate immune system acts as a first barrier against invading pathogens. It recognizes the molecular patterns of microbes with the help of highly conserved pattern-recognition receptors (PRRs). These receptors are not only activated by invading pathogens, but also by so-called “danger-associated molecular patterns” (DAMPs), which are mis-localized or altered “self” molecules. PRRs include different types of soluble and membrane-bound receptors [13, 32]. Among them are the Toll-like-receptors (TLRs). 13 mouse and 10 human TLRs have been discovered that can recognize a wide spectrum of microbial or host-derived molecules. TLRs are either bound to the cell membrane or localized intracellularly to the endoplasmic reticulum or the endo-lysosomal compartment. Their activation leads to the transcription of various pro-inflammatory cytokines via activation of transcription factors such as NF-κB [1].

TLR3 is localized in endosomes and is activated by host-derived or viral double-stranded (ds) RNA [15]. In previous studies, we found TLR3 activation to promote endothelial dysfunction in atherogenesis [28]. New in vitro studies suggest that TLR3 signaling may also play an important role in the development of AS. In cultivated valvular interstitial cells (VICs) activation of TLR3 with the dsRNA-analog polyIC resulted in NF-κB activation and upregulation of pro-inflammatory cytokines [38]. Furthermore, TLR3 activation in VICs led to increased expression of osteogenic proteins, such as Bone morphogenic protein-2 (BMP-2) and Transforming growth factor-b (TGF-b), responsible for the formation of extracellular calcium deposits [39].

Because these results provide strong evidence that TLR3 might play an important role in aortic valve cell biology, we sought to further investigate if the activation of TLR3 could affect AS development.

## Materials and methods

### In vivo experiments

#### Mice

10–12 week-old male C57/BL6-J (wildtype) mice were purchased from Janvier Labs, France. *TLR3*^*−/−*^mice on a C57/BL6-J background were obtained through our own breeding program. Knockouts were confirmed via PCR analyses of ear punches. All animals were maintained in a room at 22 °C, with a 12 h light/dark cycle, and received chow and drinking water ad libitum. Mice were held in cages with 4–5 animals. Randomization was ensured by arbitrarily assigning an animal to a cage. All animals in one cage were either assigned to the control or the treatment group, the ratio of control to treatment animals was 1:1. Only male mice were used for the experiments because female animals have significantly lower trans-aortic valve peak blood flow velocity at baseline and display different hemodynamic changes following wire injury.

All animal experiments were performed according to institutional guidelines, the German animal protection law and were approved by the Landesamt für Natur, Umwelt und Verbraucherschutz Nordrhein-Westfalen (81-02.04.2018.A250, 84-2.04.2013.A103 and 84-02-04-2013.A197.

#### Echocardiography

For all functional analyses, a Fujifilm Visualsonics Vevo 2100 or 3100 Ultra High Frequency Imaging Platform was used. Echocardiography was performed as previously described [20]. Briefly, the mice were anesthetized and their chest hair was removed. The aortic valve peak velocity was measured in the suprasternal view with a pulse-wave Doppler. Left ventricular (LV) function, wall thickness, and LV volume were each measured in parasternal long-axis and short-axis views in B- and M-mode images. The sonographer was blinded to the specifics of the experiments including the genotype and/or the medical treatment of the mice. All analyses were performed with Fujifilm Visualsonics VevoLab software.

#### Aortic valve injury

For the induction of a severe aortic valve stenosis in mice, standard protocols have been established in our lab: a conventional guide wire with a 15° angled tip (ASAHI INTECC MIRACLEbros 6) was inserted into the right carotid artery and then advanced into the left ventricle under echocardiographic guidance. The wire was pushed into the apex and pulled back just below the aortic valve level ten times, followed by 200 rotations of the wire at level of the aortic valve [20, 25]. The operating team alternated between mice from the intervention control group. The surgeon was unaware of the genotype or the medical treatment of the mice. Animals that did not survive the procedure were excluded from the analyses. No other exclusion or inclusion criteria were determined.

#### Medical treatment

For in vivo stimulation of TLR3, 100 µg polyIC (Sigma-Aldrich) was dissolved in 0.9% sodium chloride solution (NaCl) (Fresenius Kabi, Germany) and injected subcutaneously every day for 6 weeks (initial *n* = 20, 5 animals died during surgery). TLR3 was inhibited using the TLR3/dsRNA-complex inhibitor Compound 4a (C4a, Millipore Sigma). 27.5 µg C4a were dissolved in 200 µl phosphate buffered saline (PBS) and was injected subcutaneously. Control animals received similar amounts of NaCl or PBS, respectively (initial *n* = 40, 7 mice died during the procedure).

#### Histological analysis

The mice were euthanized via cervical dislocation six weeks after the aortic-valve injury. Hearts were isolated and blood was flushed by injecting 0.9% sodium chloride solution into the LV. Afterwards, the collected hearts were embedded in a tissue-freezing medium and sectioned (8 μm thickness). The sections were stained with hematoxylin and eosin according to standard protocols. Tissue calcification was measured using von-Kossa staining (Abcam Staining Kit). Collagen was visualized using Pico-Sirius-Red staining (Sigma Aldrich). Images were obtained using light/polarization (Sirius-Red) microscopy at 10x (Axio Observer, Zeiss, Germany). Aortic valve area, collagen, and calcium deposits were measured with Zeiss ZEN Imaging Software.

#### Immunofluorescence

CD68 staining was used to visualize macrophage infiltration into the stenotic aortic tissue. Aortic sections were fixed in acetone for 20 min, washed with PBS, and blocked with 1% bovine serum albumin (BSA) for 30 min. The primary antibody was diluted 1:100 (anti-CD68 rat IgG2a, Acris Antibodies, Germany) and incubated at 4 °C overnight. After washing with PBS, the sections were incubated with the secondary antibody, which was diluted 1:50 (Cy3 AffiniPure Donkey anti Rat IgG, Jackson ImmunoResearch Laboratories Inc), for 1 h at 4 °C. Vectashield mounting medium containing 4’,6’-diamidino-2-phenylindole (DAPI) (Vector Laboratories) was used for counterstaining of the nuclei.

Images were taken with an Axio Observer (Zeiss, Germany). The CD68 positive areas were automatically quantified with Zeiss ZEN Imaging Software.

#### Cytokine-analyses

After cervical dislocation of the mice, whole blood was drawn from the retroorbital plexus with heparin-coated glass pipettes. After centrifugation at 7500 g for 20 min at 4 °C, plasma was collected. Plasma-levels of IL-6, IL-10 and TNFα were measured using a Mesoscale V-plex “mouse-proinflammatory panel 1” Assay-Kit (MSD). Samples were measured in duplicates Assays were analyzed using a MESO Sector S 600 Plate reader (MSD).

#### Flow cytometry

For quantification of peripheral blood cells, 50 µl of whole blood was collected. Red blood cells were lysated and Fc- blockage was performed (Fc-Block, Pharmingen). After washing cells were stained with anti-B220 (BD Pharmingen), anti-CD8a (BioLegend), anti-CD11b (BD-Pharmingen), anti-CD4, anti-CD115, anti-Nk1.1, and anti-Ly6c (all ThermoFisher Scientific). To collect absolute numbers, CountBright Absolute Counting Beads (ThermoFisher Scientific) were added. Samples were measured on a FACSCanto II (BD Bioscience, Franklin Lakes, USA) and analyzed with FlowJo (Tree Star, Ashland, USA).

### In vitro experiments

#### Valvular interstitial cell culture and calcification

Valvular interstitial cells (VICs) were purchased from Lonza Bioscience. Cells were cultured in culturing medium (Dulbecco’s Modified Eagle’s Medium (DMEM, Thermo Fisher) containing 10% Fetal Bovine Serum (FBS) and 1% Penicillin/Streptomycin (P/S)) until passage six. Cells were incubated at 37 °C in a humidified 5% CO_2_ atmosphere and fed every second day. When the cells reached 80–90% confluence, they were treated with polyIC (10 µg/ml) (Sigma Aldrich) as well as 10 µg/ml compound C4a (Millipore Sigma) or both for 72 h. Calcification was induced in conditioning medium (DMEM containing 5% FBS, 1% P/S, 2 mmol/l disodium hydrogen phosphate, 50 µg/ml ascorbic acid).

#### TLR3 knockdown

VICs in passage 6 were seeded on 12-well plates for gene expression and 48-well plates for alizarin red staining. When cells reached 80–90% confluence, TLR3 siRNA (siRNA TLR3 107055: sense: GGUACCUGAAUUUGAAACGtt antisense: CGUUUCAAAUUCAGGUACCtc; siRNA TLR3 107056: sense: GGUACAUCAUGCAGUUCAAtt antisense: UUGAACUGCAUGAUGUACCtt) or scrambled siRNA (Thermo Fisher) as negative control were transfected into cells using Lipofectamine 2000 (4 µl/ml) (Thermo Fisher). The transfection concentration of siRNA against TLR3 was 10 nM. Cells were incubated at 37 °C in a humidified 5% CO_2_ atmosphere for 48 h after transfection. Subsequently, calcification was induced by changing to conditioning media.

qPCR was performed to analyze calcification using the osteogenic markers BMP2 (Hs00154192_m1) and Runx2 (Hs01047973_m1). Further, the pro-inflammatory cytokine IL6 (Hs00174131_m1) was measured. Activation and inhibition of TLR3 was measured using TaqMan primers for the respective gene (TLR3 Hs01551079_g1). Gapdh (Hs02758991_g1) was used as a housekeeping gene.

#### Valvular endothelial cell (VEC) culture

Human valvular endothelial cells were obtained from the Lonza Bioscience company.

VECs were cultured in culturing medium containing EBM-2 basal media supplemented with 5% FBS, 0.04% hydrocortisone, 0.4% human basic fibroblast growth factor (hFGF-β), 0.1% vascular endothelial growth factor (VEGF), 0.1% insulin like growth factor (R3-IGF-1), 0.1% ascorbic acid, 0.5 ml antibiotics (GA-1000), and 0.1% human epidermal growth factor (hEGF) (Lonza) until passage six. Cells were incubated at 37 °C in a humidified 5% CO_2_ air atmosphere and fed every second day. VECs in passage 6 were seeded on 12-well plates for gene expression. When the cells reached 80–90% confluence, VECs were treated with polyIC (10 µg/ml) (Sigma Aldrich) as well as 10 µg/ml compound C4a (Millipore Sigma) or both in hunger medium (without any supplements) followed by incubation of 24 h at 37 °C in a humidified 5% CO2 air atmosphere. Afterwards, cells were incubated in medium containing EBM-2- supplements: Basal media was supplemented with 5% FBS, 0.4% hFGF-B, 0.1% R3-IGF-1, 0.1% ascorbic acid, and 0.1% hEGF. After 24, 36, and 72 h, cells were harvested and RNA was isolated as described in the following.

qPCR was used to analyze endothelial as well as mesenchymal markers in VECs. Therefore, CD31 (Hs01065279_m) and von Willebrand factor (VWF) (Hs01109446_m1) were used as endothelial markers, whereas actin alpha2 (ACTA2) (Hs00426835_g1), Vimentin (Hs00418522_m), and Vascular cell adhesion protein 1 (VCAM1) (Hs00365486_m1) were used as mesenchymal markers. Activation and inhibition of TLR3 was measured using TaqMan primers for the respective gene (TLR3 Hs01551079_g1). Gapdh (Hs02758991_g1) was used as a housekeeping gene.

#### Gene expression

Cells were isolated using Trizol (Thermo Fisher) according to the manufacturer’s instructions. 200 ng of total RNA was reverse transcribed into cDNA using the High-Capacity cDNA Reverse Transcription Kit (Quiagen), according to the manufacturer’s protocol.

For amplification of specific genes, the TaqMan Gene Expression Assay (FAM) was used containing a combination of forward and reverse primers that match the cDNA sequences of the gene of interest. For data analysis, the relative levels of target gene expression were calculated by the comparative CT method (ΔΔCT Method).

#### Alizarin red S staining

Cells were fixed for 15 min in 4% paraformaldehyde (Millipore) followed by a washing step with ultrapure distilled water. Cells were then incubated with 1% alizarin red S solution (Sigma Aldrich) (pH 4.2) for 15–30 min. The dye was removed by washing twice with ultrapure distilled water. Alizarin red S staining was photographed with a Zeiss Axio Observer microscope.

#### Alamar blue assay

Alamar blue assay indicates metabolic active cells and therefore, it was used as a viability assay. The pre-mixed alamarBlue reagent (TermoFisher) was added to the cells and incubated for four hours at 37 °C. Afterwards, fluorescence (using an excitation of 560 nm and an emission at 590 nm) was measured using the plate reader Tecan Infinite M Plex.

#### Cell counts

To count the cell number and cell budding, microscopic images were divided in 4 quadrants. Cells and cell budding were counted in each section. The number of cells or cell buds are shown per field of view.

### Human aortic valve samples

Aortic valve specimens were collected from patients undergoing surgical aortic valve replacement for either severe aortic stenosis or aortic regurgitation. All patients provided written informed consent. The study protocols were approved by the local ethics committee (Lfd. Nr. 078/17).

After fixation in formaldehyde for 24 h, the valves were decalcified using Titriplex III- buffer (Merck) for 72 h, embedded in paraffin, and sectioned (4 μm thickness). The sections were stained with hematoxylin and eosin according to standard protocols.

#### Immunofluorescence

Samples were deparaffinized with xylene and slowly rehydrated by putting them into decreasing concentrations of ethanol, followed by washing steps. For antigen retrieval, the slides were boiled in 10 nM citrate buffer and then washed again with distilled water. Sections were incubated for 10 min at room temperature with PBS containing 0.25% Triton X-100 (PBS-T) to permeabilize the cells, followed by three washing steps with PBS. For blocking, slides were incubated 30 min at room temperature in 5% BSA in PBS-T and 0.3 M glycine. Afterwards, the primary antibody (20 µg/ml; anti-TLR3 antibody (Abcam)) diluted in 1% BSA PBS-T) was added and incubated overnight at 4 °C. The next day, slides were washed twice with PBS-T, followed by a one-hour incubation with the secondary antibody (Goat anti-rabbit Cy3 (Dianova)) diluted 1:100 in 1% BSA PBS-T) at room temperature. Finally, slides were mounted with DAPI and analyzed with a confocal microscope using 20x and 40x objectives.

### Statistical analyses

All data are presented as the mean ± SEM. Statistical significance was calculated by two-way ANOVA followed by a Tukey Test or a mixed-model followed by a Bonferroni Test for multiple comparisons. Two groups were compared using the Student *t* test. *P* values of 0.05 or less were considered to be statistically significant.

## Results

**Fig. 1:**
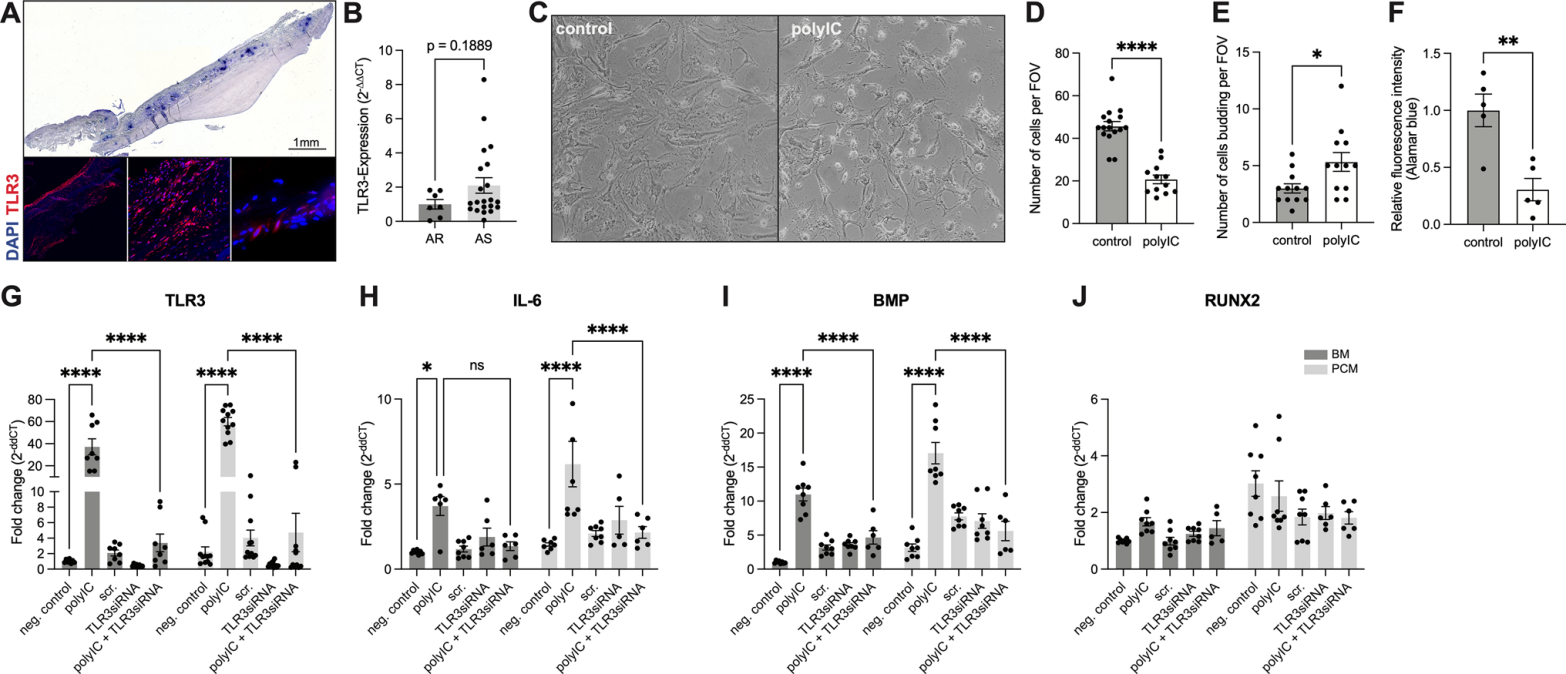
TLR3 is expressed in human aortic valves and TLR3 stimulation activates VICs: Toll-like-receptor 3 (TLR3) expression in human stenotic aortic valves (AS) was visualized via immunofluorescence staining (**A**) and quantitative PCR compared to valves explanted due to aortic valve regurgitation (AR) (**B**) (two-sided *T* test, mean ± SEM). Human valvular interstitial cells (VICs) were stimulated with the TLR3 agonist Polyinosinic:polycytidylic acid (polyIC). Representative image (**C**) and quatification of VICs upon polyIC incubation shows decreased cell numbers (**D**, number of cells per field of view (FOV)) and increased cell budding (**E**). Cell viability was quantified with AlamarBlue assay (**F**) (****p < 0.0001, **p < 0.01, *p < 0.05, two-sided *T* test, mean ± SEM). For qPCR analysis, VICs were cultured either in basal medium (BM) or pro-calcifying medium (PCM) and TLR3 knockdown was performed with TLR3-siRNA, scramble-si RNA (scr.) was used as control. qPCR analysis of TLR3, interleukin-6 (IL-6), bone-morphogenic protein (BMP) and runt-related transcription factor 2 (RUNX2) (**G**–**J**). (****p < 0.0001, two-way ANOVA with Tukey multiple comparisons test, mean ± SEM)

Surgically explanted human stenotic aortic valves were analyzed for TLR3 expression in diseased AS tissue. Using immunohistochemistry, TLR3 was detected in all three layers of the aortic valve (Fig. 1A). Quantitative real-time PCR analyses of explanted human aortic valves confirmed these results. TLR3 expression levels in AS samples where slightly increased when compared to valves that were explanted due to severe aortic valve regurgitation (AR), because of the heterogeneity of the analyzed specimens these results were not significant (Fig. 1B).

In vitro experiments have suggested that TLR3 mediates calcification and inflammation in isolated VICs [38, 39]. To confirm these results, we stimulated cultured VICs with polyIC. Microscopic images of these polyIC-treated cells (Fig. 1C) showed clear signs of reduced viability (Fig. 1F); the cells were fewer in number (Fig. 1D) and displayed increased budding compared (Fig. 1E) to control cells. Gene expression analyses showed a strong induction of TLR3 in VICs after polyIC stimulation and increased expression of pro-inflammatory and osteogenic genes. The mRNA levels of IL-6 and BMP2 were significantly elevated in polyIC-treated cells. This response is likely TLR3-dependent, since TLR3 knockdown with siRNA prevented this pro-inflammatory and osteogenic reaction. Interestingly, these effects were independent from the type of medium used. Cells cultured in pro-calcifying medium (PCM) showed a similar response upon polyIC treatment as cells cultured in basal medium (BM). The expression of *RUNX2*, a gene that also stimulates osteoblastic differentiation, was unchanged upon polyIC stimulation in VICs (Fig. 1G–J).

**Fig. 2:**
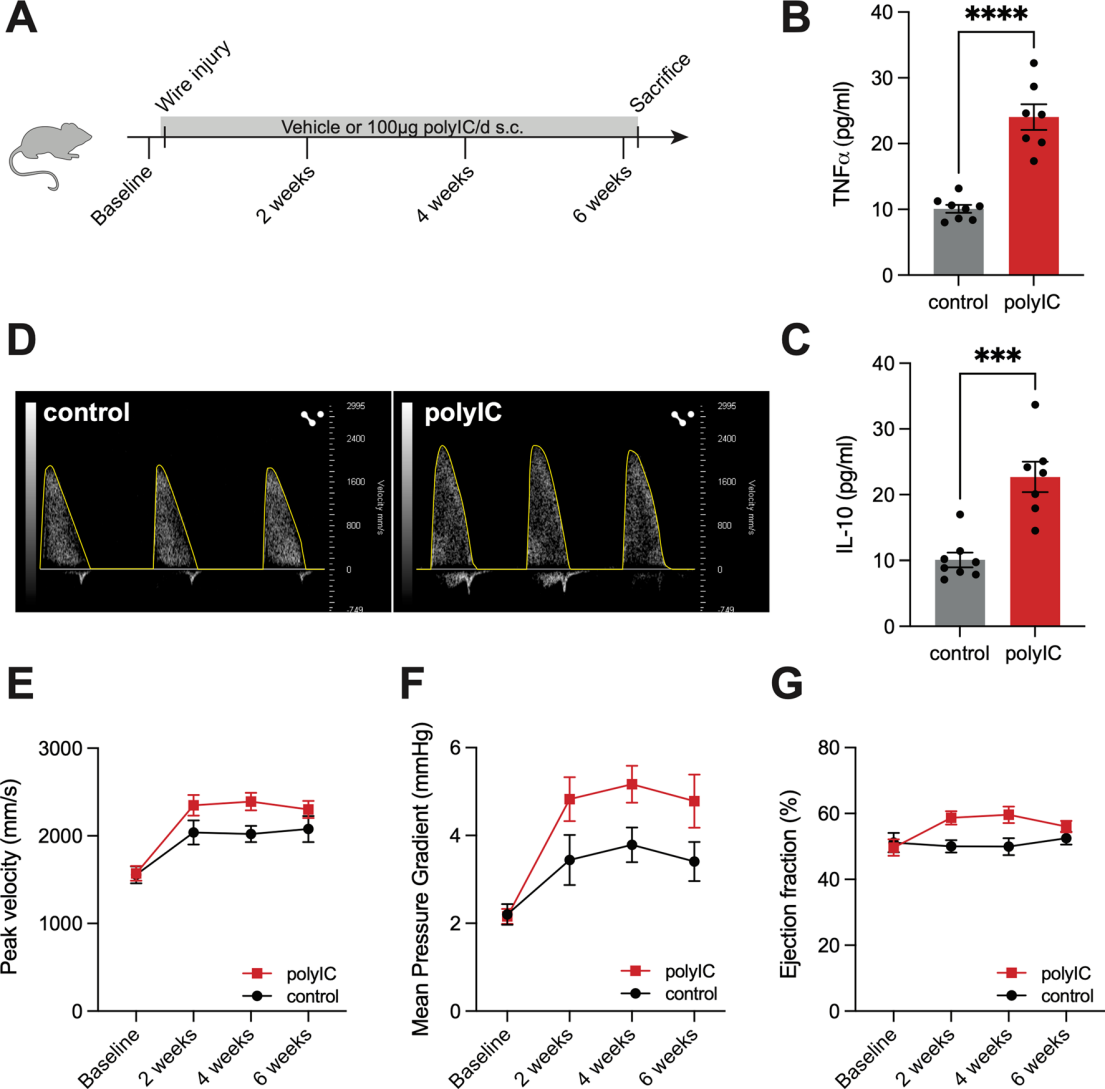
TLR3 stimulation enhances AS development in mice: AS was induced in C57BL6/J mice via wire injury. The mice received daily injections of polyIC or vehicle control (NaCl) respectively. Ultrasound was performed every two weeks after surgery (**A**). Serum concentration of pro- and anti-inflammatory cytokines after 6 weeks (**B**, **C**). Peak velocity levels (**D**, **E**) and mean pressure gradient (**F**) over the aortic valve was minimally increased upon polyIC stimulation, left ventricular function remained unchanged (**G**) (*n* = 7–8). (****p < 0.0001, ***p < 0.001, mixed-effect model with Bonferroni multiple comparisons test (**E**–**G**) or two-sided *T* tests (**B**, **C**), mean ± SEM)

Because AS development is not directly linked to a single cellular response but is rather the result of complex interaction between resident and non-resident effectors, we next studied the potential role of TLR3 in an in vivo mouse model of AS. For this, AS was induced in wildtype mice via direct injury of the aortic valve with a coronary wire that was introduced into the left ventricle under echocardiographic guidance and rotated within the aortic valve. TLR3 activation was achieved with daily injections of 100 µg polyIC over a period of 6 weeks after surgical induction of AS. Control animals were treated with vehicle control (0.9% NaCl solution). Ultrasound examinations were performed after 2, 4, and 6 weeks and were used to assess the functional development of AS (Fig. 2A). Serum levels of cytokines such as TNFα were upregulated in the polyIC-treated mice at the time of sacrifice, confirming the pro-inflammatory effect of the treatment. Interestingly, the levels of IL-10, a mainly anti-inflammatory cytokine were also increased in polyIC-treated mice (Fig. 2B, C). The trans-aortic-valve peak velocities of polyIC-treated mice were marginally elevated compared to control mice but did not reach statistical significance (Fig. 2D, E, Supplemental Table 1). In the same fashion, the mean pressure gradient over the aortic valve of polyIC-treated animals was increased compared to the vehicle-treated group (Fig. 2F). The left-ventricular function of both groups remained unchanged (Fig. 2G). Four potential mechanisms may be responsible for the marginal effects seen in this model. First, the mouse model is independent of TLR3-activation. Second, the model is already driven by endogenous TLR3 ligands, which saturate the effect. Third, the dose of polyIC could be insufficient. Fourth, overstimulation of TLR3 with polyIC leads to a chronic inflammatory state, which is not a good reflection of the human pathology of AS.

**Fig. 3:**
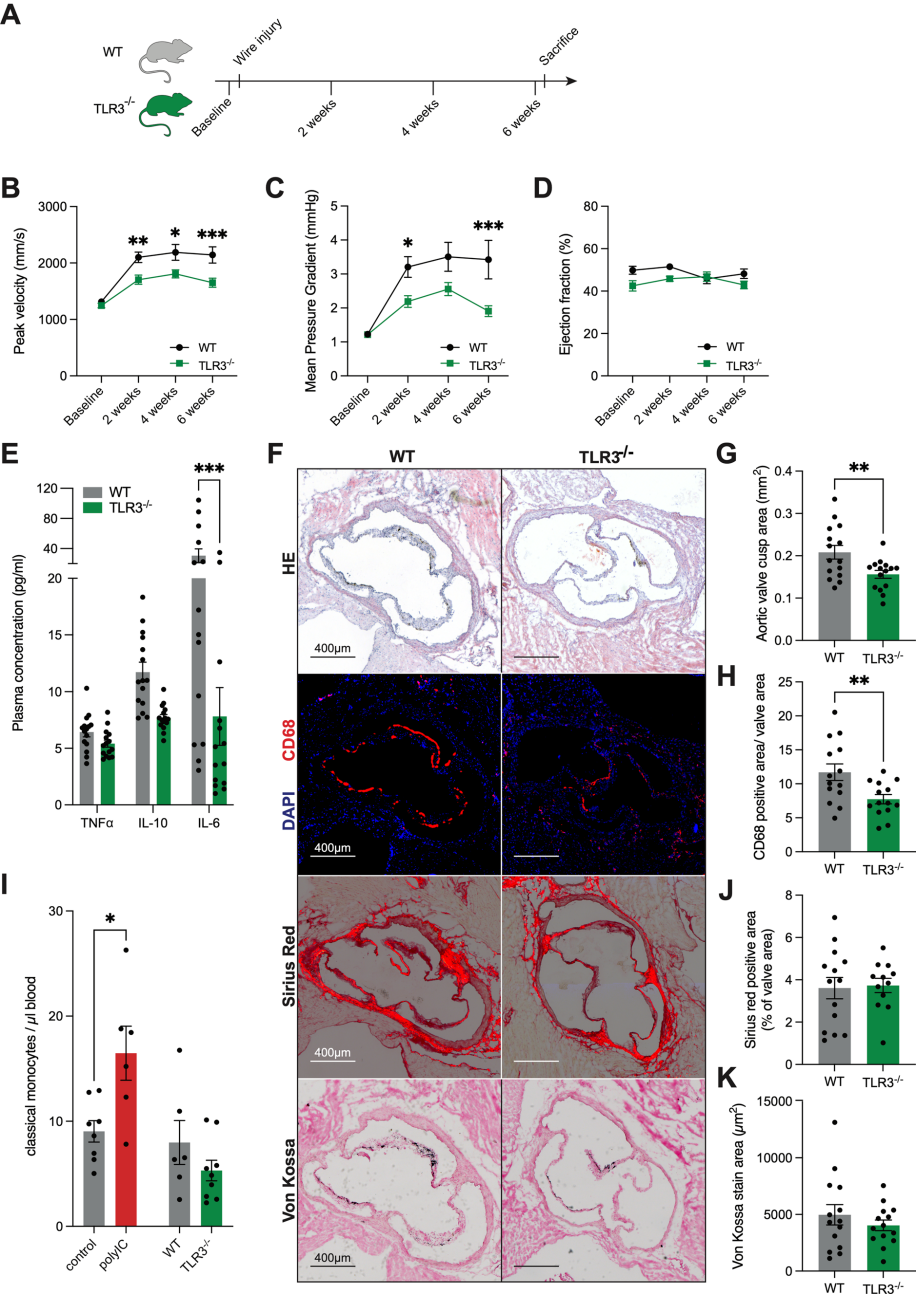
AS development is impaired in TLR3-deficient mice: AS was induced in WT and TLR3^−/−^ mice via mechanical wire injury (**A**). Trans-aortic valve blood flow peak velocity (**B**) and mean gradient (**C**) did not increase in the same degree in TLR3^−/−^ animals as in wildtype mice after surgery. Left ventricular function remained unchanged in both groups (**D**) (*n* = 19–20, *** *p* < 0.001, **p < 0.01, *p < 0.05, mixed-effect model with Bonferroni multiple comparisons test, mean ± SEM). Plasma concentrations of pro- and anti-inflammatory cytokines (**E**). Histologic analysis of the aortic valve (**F**): H.E.-staining with quantitative analysis of the valve cusp area (**G**), CD68 staining: intra-valvular monocyte infiltration (**H**). FACS analysis of monocytes in peripheral blood of polyIC-treated mice (experimental design in Fig. 2A) and TLR3^−/−^ mice vs. control (**I**) (*p < 0.05, two-sided *T* tests, mean ± SEM). Quantification of valvular fibrosis (**J**) and calcification (**K**). (*n* = 14–15, **p < 0.01, two-sided *T* tests, mean ± SEM)

To address these confounders, we first investigated if baseline endogenous activation of TLR3 contributes to AS development in our in vivo model. For this, TLR3 knockout mice were examined and compared to wildtype (WT) mice. In both groups, AS was induced via a direct wire injury, as described above, no additional pharmaceutical intervention was performed (Fig. 3A). Ultrasound examinations were performed every 2 weeks and indicated that TLR3-deficient mice failed to develop AS to a similar degree as WT mice after wire injury. The trans-aortic-valve peak velocity in the TLR3 knockout mice was significantly decreased compared to WT animals after two, four, and six weeks (Fig. 3B, Supplemental Table 2). In the same manner, the mean pressure gradient over the valve was significantly lower in TLR3^−/−^ mice (Fig. 3C). A decline in left-ventricular function as a cause for the lower peak velocities could be excluded (Fig. 3D). Interestingly, the serum concentration of IL-6 was decreased in TLR3^−/−^ mice suggesting a reduction of the endogenous pro-inflammatory state driving disease development (Fig. 3E). Six weeks after surgery, the mice were sacrificed and the hearts were explanted for histological analyses (Fig. 3F). The area of the aortic valve, as a marker for valve thickening, was determined in HE-stained sections. The valve cusp area of TLR3^−/−^ mice was significantly smaller than that of WT mice (Fig. 3G). TLR3 deletion also went along with reduced intra-valvular inflammation upon wire injury. Immunofluorescence staining of the monocyte marker, CD68, showed a reduced invasion of monocytes into valves of TLR3^−/−^ mice compared to WT mice (Fig. 3H). A similar effect was detected in peripheral blood-cell counts. The increase in circulating classical monocytes observed in polyIC-treated mice was not evident in TLR3^−/−^ mice (Fig. 3I). Nevertheless, this intra-valvular and systemic inflammatory reaction did not correlate with increased fibrosis or calcification of the aortic valve tissue. Sirius red (collagen) and von Kossa staining (calcium deposits) showed no significant differences (Fig. 3J, K).

**Fig. 4:**
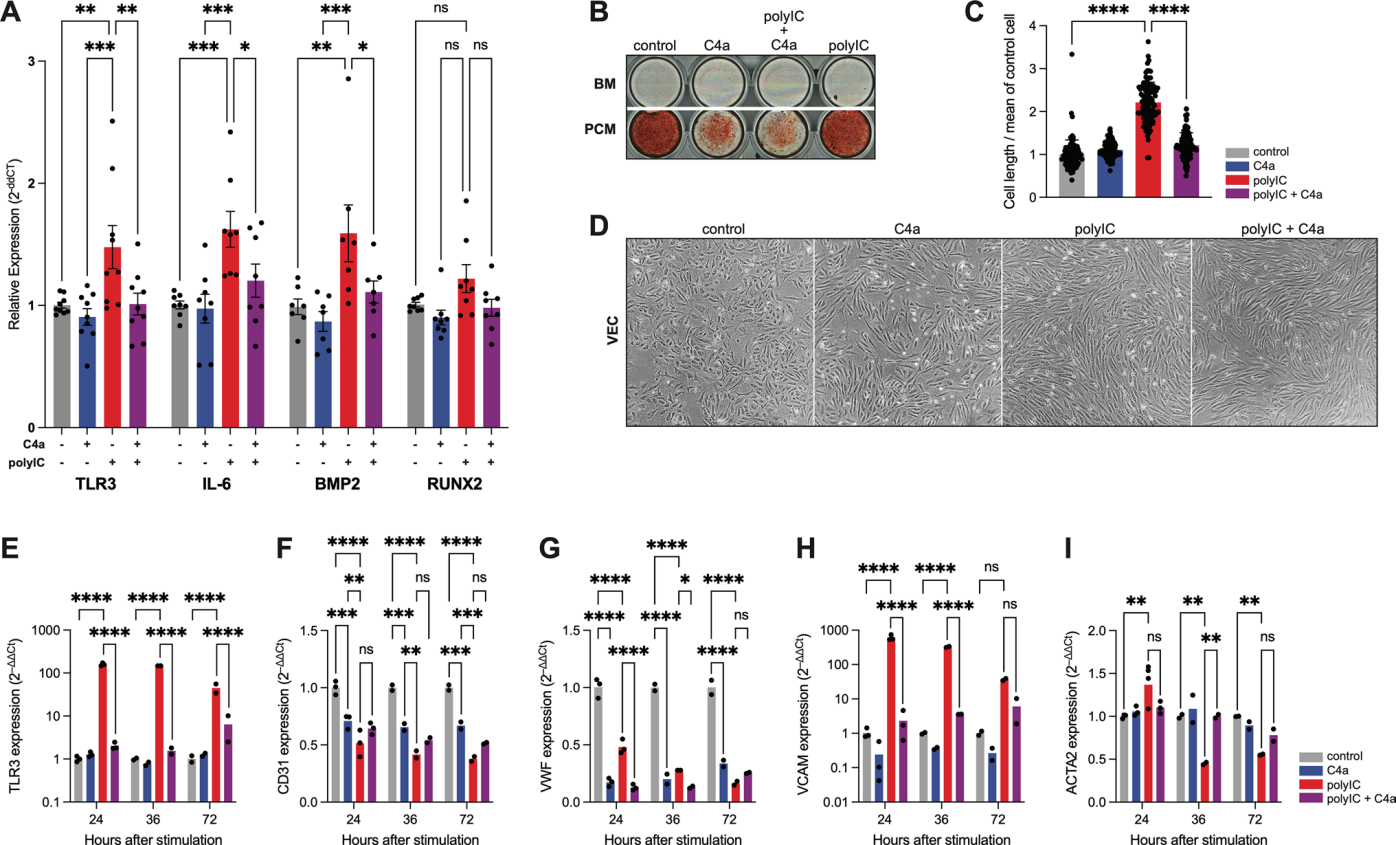
C4a reduces inflammation and calcification in valvular cells: Valvular interstitial cells (VICs) were treated with polyIC and/or C4a. TLR3 inhibition prevented upregulation of TLR3, IL-6 and BMP. No significant difference could be observed for RUNX2 (**A**) (***p < 0.001, **p < 0.01, *p < 0.05, two-way ANOVA with Tukey multiple comparisons test, mean ± SEM). Extracellular calcium deposits visualized by Alizarin red S staining in VIC cultivated in basal medium (BM) or pro-calcifying medium (PCM). Concomitant C4a incubation prevented VIC calcification upon polyIC stimulation (**B**). Relative length of valvular endothelial cells (VECs) and microscopic images 24 h after incubation with polyIC ± C4a (**C**, **D**). mRNA expression of TLR3 (**E**), CD31 (**F**), von Willebrand factor (VWF) (**G**), vascular cell adhesion molecule 1 (VCAM1) (**H**), alpha actin 2 (ACTA) (**I**) by VEC following stimulation with polyIC ± C4a (****p < 0.0001, ***p < 0.001, **p < 0.01, *p < 0.05, two-way ANOVA with Tukey multiple comparisons test, mean ± SEM)

These results suggested that endogenous TLR3 activation contributes to AS in mice and raised the question of whether pharmacological inhibition of TLR3 could be used to prevent the development of AS. A small molecule named Compound 4a (C4a) inhibits the formation of the complex between TLR3 and double-stranded RNA and can suppress the expression of proinflammatory cytokines in immortalized macrophages [4]. We investigated the efficacy of C4a in VICs under standard conditions and upon stimulation with polyIC. Cells incubated with C4a showed reduced expression of TLR3, the pro-inflammatory cytokine IL6, and the pro-osteogenic cytokine BMP2 (Fig. 4A). Additionally, the formation of macroscopic calcium depositions was significantly reduced by C4a treatment in VICs (Fig. 4B).

Not only VICs, but also valvular endothelial cells (VECs) have been attributed to the development of AS [11, 27]. We therefore studied the effects of TLR3 stimulation and inhibition in these cells. Incubation of VECs with polyIC resulted in the transformation to a more elongated spindle-shaped phenotype associated with endothelial-mesenchymal transition (EndoMT) [14]. The TLR3 antagonist C4a slightly diminished this change (Fig. 4C, D). To further elucidate this observation, we quantified the expression of genes affected by EndoMT. Importantly, the upregulation of TLR3 via polyIC was clearly evident in VECs (Fig. 4E). The endothelial markers CD31 and von Willebrand factor (VWF) where significantly downregulated while the endothelial activation marker vascular cell adhesion molecule 1 (VCAM1) was upregulated upon polyIC incubation (Fig. 4F–H). Interestingly, actin alpha 2 (ACTA2) showed time-dependent differences with an increased expression after 24 h followed by a subsequent downregulation (Fig. 4I). TLR3 inhibition with C4a nullified the effects on TLR3, VCAM1 and ACAT2 expression, but showed dramatic effects on CD31 and VWF without additional polyIC stimulation (Fig. 4F–I).

**Fig. 5:**
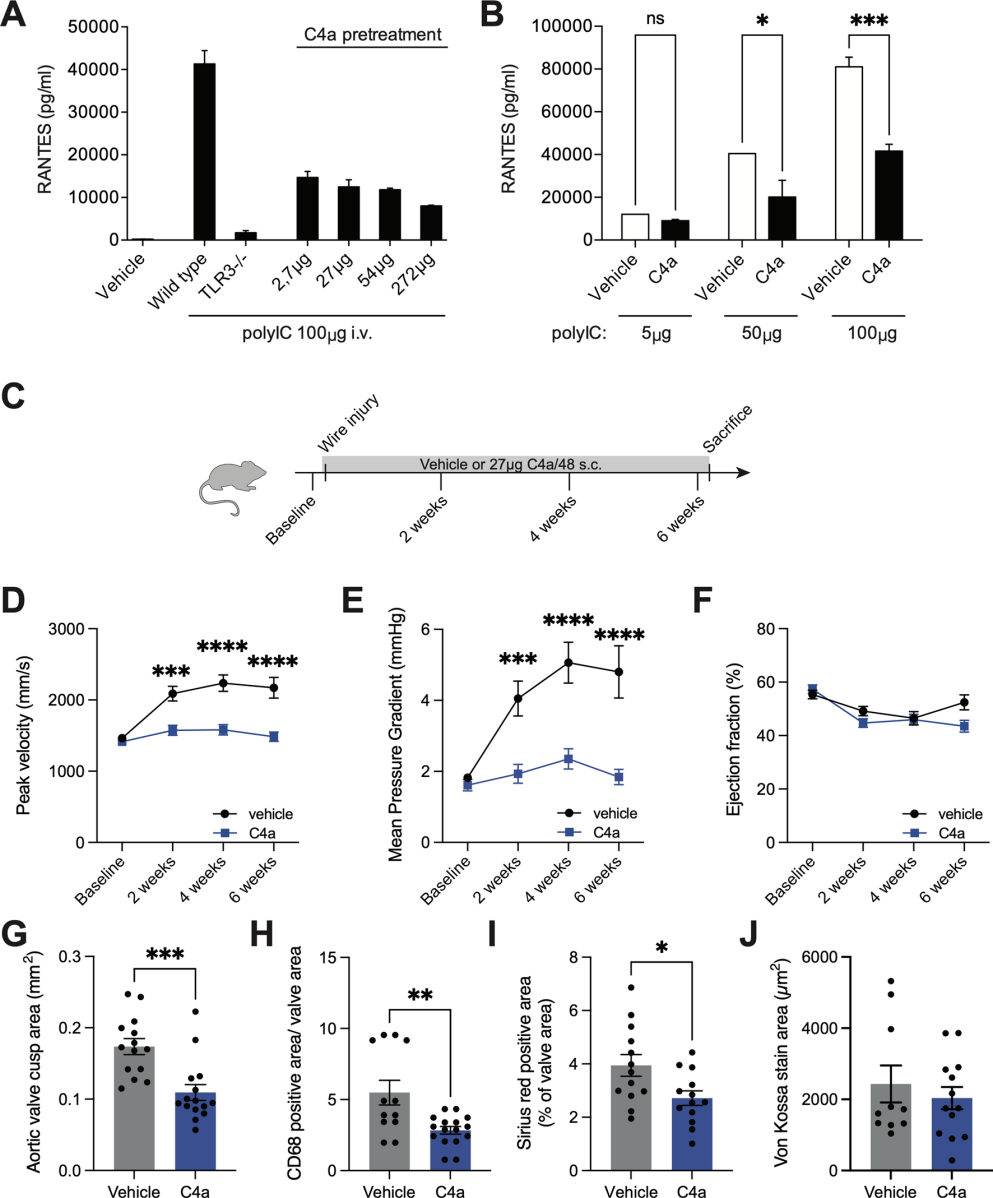
Pharmacological TLR3 inhibition reduces AS formation in mice: Serum concentration of Regulated on activation, normal T cell expressed and secreted (RANTES) in mice 6 h after 100 µg polyIC injection and concomitant dose escalation C4a treatment (**A**) (*n* = 2). RANTES serum concentration after C4a treatment and injection of increasing polyIC dose (**B**) (***p < 0.001, *p < 0.05, two-way ANOVA with Tukey multiple comparisons test, mean ± SEM, *n* = 3). C4a or vehicle was injected in WT mice every second day after AS induction (*n* = 20) (**C**). C4a inhibited increase in blood flow peak velocity (**D**) and mean gradient (**E**) compared to vehicle-treated mice. Left ventricular ejection fraction remained unchanged (**F**) (**** *p* < 0.0001, ***p < 0.001, mixed-effect model with Bonferroni multiple comparisons test, mean ± SEM). Aortic valve cusp area (**G**), infiltration of CD68 positive cells (**H**) and fibrosis (**J**) was significantly increased in C4a treated mice. Calcification was similar in both groups (**H**) (*n* = 12–16, ***p < 0.001, **p < 0.01, *p < 0.05, two-sided *T* tests, mean

To determine the correct dosage in our in vivo mouse model, we tested the anti-inflammatory potential of C4a in WT and TLR3^−/−^ mice following a single injection of polyIC. Serum levels of a TLR3-dependent cytokine RANTES (Regulated on Activation, Normal T cell Expressed and Secreted; chemokine ligand 5, CCL5) were measured 6 hours after the injection. As expected, the WT mice reacted with a strong secretion of RANTES, whereas TLR3 knockout mice did not (Fig. 5A). Pretreatment of WT mice with C4a significantly reduced the release of RANTES into the circulation in a dose-dependent manner. Even after four hours (time of maximum inflammatory response to polyIC), pretreatment of mice with 27 μg C4a greatly impaired the production of RANTES (Fig. 5B).

These data demonstrate that C4a effectively inhibits a TLR3-mediated pro-inflammatory response in mice and prompted us to investigate TLR3 antagonism with C4a in the wire-induced model of aortic valve stenosis. WT mice were treated with 27 µg C4a every other day following aortic valve injury. Littermates received injections of vehicle (PBS) as a control (Fig. 5A). Echocardiography was performed after 2, 4, and 6 weeks, which showed reduced AS development in C4a-treated mice. Peak velocity and mean gradient of the aortic valve remained stable after wire injury in C4a-treated mice–in contrast to control mice that developed AS (Fig. 5D, E, Supplemental Table 3). Again, left-ventricular function remained unchanged between the two groups (Fig. 5F). Histological sections confirmed that the C4a-treated mice failed to develop AS after wire injury. The aortic-valve cusp area was significantly smaller in C4a-treated mice, which goes along with a reduction in macrophage infiltration in the valve cusps, confirming the anti-inflammatory effect of C4a (Fig. 5G, H). Valves from the treatment group showed fewer signs of fibrosis–the area of the valve that was positive for Sirius red was noticeably smaller in mice that received C4a injections (Fig. 5I). However, calcification, as a marker for late stages of AS, was not reduced in C4a mice compared to the control group (Fig. 5J).

## Discussion

In this study, we demonstrate that TLR3-mediated signaling contributes to AS development. TLR3 is expressed in human stenotic valves and TLR3 stimulation leads to increased expression of pro-inflammatory and pro-osteogenic markers in VICs. While, subjecting mice to exogenous TLR3 stimulation with polyIC only slightly enhanced AS development, TLR3-deficient mice did not develop AS after wire injury. Pharmacological inhibition of TLR3 with the small molecule C4a, did not only suppress the pro-inflammatory and pro-osteogenic effects in human VICs and VECs in vitro, but also suppressed AS development in vivo. C4a treatment prevented AS development in mice after wire injury.

Toll-like receptors belong to a genetically conserved family of membrane-bound receptors, each of which recognize a specific set of molecular patterns. So far, 10 human TLRs and 13 mammalian TLRs have been described [9]. While most of these TLRs are located on the plasma membrane to recognize extracellular pathogens, TLR3 (along with TLR7, -8, and -9) is located in the endolysosomal compartment, so that it can detect intracellular pathogens. Upon simulation with their ligand, TLRs dimerize and activate common signaling pathways with the help of the adaptor protein MyD88. In contrast to the other TLRs, TLR3 signaling exclusively uses the adaptor protein TRIF for its downstream signaling–MyD88 is not necessary for TLR3 activation. Both routes lead to the expression of pro-inflammatory cytokines via NF-κB and the expression of antiviral molecules via interferon regulatory factor (IRF) [1, 9, 15]. TLR3 is activated by double-stranded RNA (dsRNA), which comes from viruses [2, 41]. Also host-derived dsRNA, such as micro RNAs, that are released upon cellular damage or necrosis have been shown to activate TLR3 [3, 5, 33, 42]. In experimental setups, polyIC is a synthetic mimic for double-stranded RNA and can be used for TLR3 activation [2]. TLRs are crucial to cell homeostasis and cellular defense against pathogens, however, inappropriate activation can lead to chronic inflammatory syndromes. In the cardiovascular system, TLR3 activation has been linked to atherosclerosis. Our group has shown that activation of TLR3 impaired endothelial function in mice [28]. Similarly, *TLR3*^*−/−*^ myeloid chimeric *LDLR*^*−/−*^ mice had significantly reduced aortic inflammation, as well as atherosclerotic plaque size [17].

Recent publications have suggested that TLR3 might also play an important role in the development of AS. Expression analysis has shown that most TLRs are expressed in VICs from healthy donors and in VICs from AS patients. VICs stimulated with specific ligands of different TLRs, especially TLR3 stimulation with viral RNA, led a strong activation of NF-κB [16]. Furthermore, polyIC stimulation of VICs isolated from stenotic aortic valves led to increased production of BMP-2, transforming growth factor beta-1 (TGF-β1), and alkaline phosphatase (ALP). This resulted in the formation of calcium deposits by VICs. siRNA knockdown of TLR3 abrogated this effect [39]. TLR3 stimulation in VICs has not only been linked to an increased pro-osteogenic effect but also to an enhanced inflammatory response. PolyIC stimulation of VICs increased the production of IL-6, IL-8, monocyte chemoattractant protein-1, and ICAM-1. Again, inhibition of TLR3 or NF-κB was able to abolish this effect [38].

Besides TLR3, other TLRs have also been linked to AS. Expression analysis of VICs from stenotic valves have shown that most TLRs are expressed, TLR4 being the most abundant receptor [16, 19]. TLR4 recognizes lipopolysaccharid (LPS) derived from gram negative bacteria [1]. LPS stimulation of VICs has been shown to induce a strong pro-inflammatory reaction via NF-kB signaling [16, 19]. VICs from stenotic valves are more susceptible to LPS treatment than those from health donors [8, 37]. In the same fashion TLR2 signaling upon stimulation with peptidoglycans from gram-positive bacteria promotes a pro-osteogenic reaction in VICs [16]. TLR2 and 4 classically recognize bacterial ligands [1] and recurrent low grade bacterial infections have been linked to AS[29] but also sterile activators of those TLRs have been found to be relevant in AS development. For example, Biglycan[30], a proteoglycan, or the nuclear protein High Mobility Group Box 1 (HMGB1), when secreted extracellularly can induce sterile inflammation and calcification in VICs via TLR2/4 signaling [35]. It has been shown that this pro-inflammatory response upon TLR 2 and 4 can be antagonized by the anti-inflammatory Interleuktin-37 (IL-37). Notably IL-37 only affects MyD88 dependent TLR signaling, therefore TLR3 mediated inflammation is not affected [40]. So far, in vivo relevance of these signaling pathways has not been investigated.

This study advocates the concept that inhibition of TLR3 might be a potential therapeutic intervention to counteract TLR3 overactivation in the pathogenesis of AS. Because of its intracellular localization, TLR3 inhibition has thus far been challenging. But in 2011, Cheng et al. have identified the small molecule Compound 4a as a specific inhibitor of TLR3. C4a binds directly to TLR3 and prevents the formation of the complex between dsRNA and TLR3. TLR3 activation and its downstream signaling effects are blocked by C4a. Other TLRs are not affected by C4a and it has been shown to inhibit the production of pro-inflammatory cytokines upon polyIC stimulation in vitro [4]*.* In vivo, C4a has been shown to protect mice from radiation-induced gastrointestinal syndrome, a process that is considered to be primarily dependent on TLR3 signaling [31].

Several limitations of the study must be considered when interpreting the data. First, the injury-induced mouse model of AS is an artificial model and only reflects parts of the human pathology. Cusp thickening, infiltration of immune cells, and remodeling of the extracellular matrix occur within six weeks upon wire injury whereas human AS develops over several years. Second, only male mice were used for the experiments and therefore we cannot account for gender specific effects contributing to AS development. Third, the human VIC and VEC cell lines were purchased commercially and derive from donors whose medical records are unknown to us. Therefore, cardiovascular comorbidities and other unknown external factors may have influenced the results.

Nevertheless, this study is the first to demonstrate a significant role of TLR3 signaling in AS development both in vitro and in vivo. Because of the profound efficacy of C4a in preventing AS development in mice, targeting TLR3 in humans might be a viable therapeutic option to protect against the development of AS. Further studies in large animal models are needed to elucidate the safety and effectiveness of C4a treatment before its use in humans can be considered.

### Supplementary Information

Below is the link to the electronic supplementary material.

Supplementary file1 (DOCX 62 KB)

## Data Availability

All data supporting the findings of this study are available within the article and its supplementary material. Any additional information can be requested by contacting the corresponding author.
