# Supplementary material for: Toll-like receptor-3 contributes to the development of aortic valve stenosis
Source: Basic Res Cardiol. 2023 Feb 1;118(1):6. doi: 10.1007/s00395-023-00980-9 (PMC9892139; doi:10.1007/s00395-023-00980-9)
Supplement: Supplementary file 1 — Supplementary file1 (DOCX 62 KB) [file 395_2023_980_MOESM1_ESM.docx]

**Supplemental Table 1:**

Echocardiographic parameters of WT mice treated with polyIC or vehicle control (NaCL) at baseline and 2, 4, and 6 weeks following wire-injury of the aortic valve as illustrated in Figure 2, A.

|  |  | **Baseline** | | | | **Week 2** | | | | **Week 4** | | | | **Week 6** | | | |  |
| --- | --- | --- | --- | --- | --- | --- | --- | --- | --- | --- | --- | --- | --- | --- | --- | --- | --- | --- |
| Parameter | Value | Control | | PolyIC | | Control | | PolyIC | | Control | | PolyIC | | Control | | PolyIC | | P value |
| **Suprasternal Axis** | | | | | | | | | | | | | | | | | | |
| VTI | mm | 72,16 | (24,66) | 72,83 | (16,48) | 72,43 | (24,04) | 91,85 | (21,16) | 92,00 | (37,05) | 99,84 | (31,16) | 65,55 | (17,43) | 104,20 | (60,75) | **0,042** |
| Mean Vel | mm/s | 731,52 | (101,12) | 729,53 | (91,30) | 893,94 | (263,89) | 1089,18 | (154,89) | 963,68 | (145,36) | 1130,77 | (126,87) | 907,71 | (179,88) | 1079,54 | (148,45) | 0,093 |
| Mean Grad | mmHg | 2,20 | (0,63) | 2,16 | (0,54) | 3,44 | (1,62) | 4,83 | (1,32) | 3,79 | (1,12) | 5,17 | (1,11) | 3,41 | (1,26) | 4,78 | (1,10) | 0,086 |
| Peak Vel | mm/s | 1525,40 | (226,63) | 1571,04 | (195,47) | 2038,94 | (389,02) | 2349,42 | (310,03) | 2022,06 | (261,93) | 2390,85 | (265,65) | 2078,12 | (421,89) | 2301,75 | (82,59) | 0,246 |
| Peak Grad | mmHg | 9,54 | (2,94) | 10,01 | (2,52) | 17,16 | (6,06) | 22,41 | (5,70) | 16,60 | (3,95) | 23,11 | (5,03) | 17,90 | (6,71) | 21,42 | (1,38) | 0,272 |
| **Parasternal short axis** | | | | | | | | | | | | | | | | | | |
| Heart Rate | BPM | 451,94 | (19,66) | 427,10 | (47,44) | 449,97 | (55,03) | 435,23 | (43,03) | 448,69 | (42,38) | 464,29 | (66,47) | 481,02 | (43,73) | 490,74 | (29,03) | 0,625 |
| Diameter;s | mm | 3,00 | (0,38) | 3,10 | (0,37) | 2,91 | (0,25) | 2,58 | (0,42) | 3,10 | (0,33) | 2,64 | (0,30) | 3,09 | (0,26) | 2,80 | (0,09) | 0,089 |
| Diameter;d | mm | 4,04 | (0,19) | 4,06 | (0,23) | 3,88 | (0,21) | 3,70 | (0,45) | 4,14 | (0,27) | 3,84 | (0,22) | 4,21 | (0,19) | 3,93 | (0,12) | 0,071 |
| Volume;s | uL | 35,94 | (9,59) | 38,80 | (10,30) | 32,87 | (7,12) | 25,11 | (10,26) | 38,65 | (10,16) | 26,16 | (7,76) | 37,98 | (8,11) | 30,28 | (2,35) | 0,129 |
| Volume;d | uL | 72,19 | (7,76) | 72,79 | (9,35) | 65,30 | (8,46) | 59,17 | (16,62) | 76,41 | (11,91) | 63,89 | (9,00) | 79,37 | (8,77) | 67,76 | (4,50) | 0,091 |
| Stroke Volume | uL | 36,24 | (2,21) | 33,99 | (5,12) | 32,43 | (3,37) | 34,07 | (7,04) | 37,76 | (5,53) | 37,73 | (3,52) | 41,39 | (3,73) | 37,48 | (3,14) | 0,132 |
| Ejection Fraction | % | 51,18 | (9,72) | 47,50 | (9,82) | 50,04 | (5,35) | 58,68 | (5,42) | 49,96 | (7,26) | 59,62 | (6,62) | 52,49 | (5,48) | 56,01 | (2,41) | 0,204 |
| Fractional Shortening | % | 26,05 | (6,50) | 23,80 | (5,97) | 25,06 | (3,25) | 30,51 | (3,37) | 25,21 | (4,38) | 31,33 | (4,48) | 26,77 | (3,35) | 28,83 | (1,57) | 0,225 |
| Cardiac Output | mL/min | 16,40 | (1,18) | 14,61 | (3,08) | 14,65 | (2,64) | 14,79 | (3,11) | 16,93 | (2,99) | 17,46 | (2,55) | 19,87 | (2,21) | 18,38 | (2,52) | 0,271 |
| LV Mass | mg | 119,11 | (7,52) | 115,46 | (21,60) | 110,04 | (33,21) | 108,37 | (8,15) | 127,55 | (18,56) | 131,02 | (22,61) | 133,54 | (24,12) | 121,95 | (1,91) | 0,277 |
| LV Mass Cor | mg | 95,29 | (6,02) | 92,37 | (17,28) | 88,03 | (26,56) | 86,70 | (6,52) | 102,04 | (14,85) | 104,82 | (18,09) | 106,84 | (19,30) | 97,56 | (1,53) | 0,277 |
| LVAW;s | mm | 1,29 | (0,17) | 1,20 | (0,17) | 1,19 | (0,25) | 1,37 | (0,19) | 1,30 | (0,21) | 1,49 | (0,23) | 1,36 | (0,22) | 1,27 | (0,23) | 0,406 |
| LVAW;d | mm | 0,91 | (0,11) | 0,87 | (0,18) | 0,92 | (0,23) | 0,98 | (0,14) | 0,96 | (0,19) | 1,08 | (0,12) | 0,98 | (0,18) | 0,95 | (0,10) | 0,715 |
| LVPW;s | mm | 0,93 | (0,10) | 0,89 | (0,10) | 0,88 | (0,09) | 0,96 | (0,19) | 0,93 | (0,13) | 1,04 | (0,10) | 0,93 | (0,06) | 1,04 | (0,17) | 0,051 |
| LVPW;d | mm | 0,68 | (0,04) | 0,67 | (0,07) | 0,64 | (0,07) | 0,69 | (0,17) | 0,67 | (0,07) | 0,73 | (0,07) | 0,67 | (0,07) | 0,73 | (0,15) | 0,197 |
| **Parasternal long axis** | | | | | | | | | | | | | | | | | | |
| Heart Rate | BPM | 457,60 | (20,40) | 437,90 | (39,77) | 451,26 | (53,88) | 527,89 | (201,37) | 460,54 | (37,03) | 464,78 | (67,02) | 490,07 | (39,07) | 488,22 | (23,09) | 0,915 |
| Area | mm2 | 16,97 | (1,54) | 19,01 | (4,22) | 18,80 | (2,50) | 15,17 | (3,79) | 17,21 | (2,29) | 14,86 | (2,01) | 16,17 | (2,71) | 17,15 | (1,66) | 0,537 |
| Area;s | mm2 | 16,97 | (1,54) | 18,03 | (2,09) | 18,80 | (2,50) | 15,17 | (3,79) | 17,21 | (2,29) | 14,86 | (2,01) | 16,17 | (2,71) | 17,15 | (1,66) | 0,537 |
| Area;d | mm2 | 26,40 | (2,74) | 27,61 | (2,37) | 27,97 | (1,82) | 23,56 | (5,24) | 25,27 | (2,46) | 25,01 | (1,79) | 26,44 | (3,42) | 24,31 | (0,72) | 0,200 |
| Volume | uL | 34,07 | (6,56) | 42,28 | (16,47) | 39,27 | (7,57) | 27,28 | (11,30) | 36,62 | (7,35) | 27,55 | (6,79) | 33,35 | (8,83) | 36,13 | (5,78) | 0,627 |
| Volume;s | uL | 34,07 | (6,56) | 38,20 | (8,11) | 39,27 | (7,57) | 27,28 | (11,30) | 36,62 | (7,35) | 27,55 | (6,79) | 33,35 | (8,83) | 36,13 | (5,78) | 0,627 |
| Volume;d | uL | 72,40 | (14,39) | 78,26 | (11,58) | 76,84 | (6,57) | 58,76 | (20,98) | 71,17 | (11,04) | 68,56 | (8,96) | 75,57 | (15,23) | 65,78 | (2,00) | 0,204 |
| Stroke Volume | uL | 38,33 | (9,75) | 40,06 | (5,28) | 37,57 | (5,11) | 31,48 | (10,12) | 34,55 | (4,79) | 41,00 | (4,18) | 42,22 | (10,18) | 29,64 | (6,29) | **0,027** |
| Ejection Fraction | % | 52,90 | (6,34) | 51,47 | (4,79) | 49,10 | (7,14) | 54,23 | (3,93) | 48,77 | (4,00) | 60,18 | (5,13) | 55,73 | (7,55) | 45,46 | (10,51) | 0,064 |
| Fractional Shortening | % | 10,82 | (4,06) | 11,42 | (3,08) | 8,83 | (6,62) | 10,88 | (3,55) | 9,76 | (3,62) | 13,11 | (5,52) | 14,70 | (4,46) | 8,33 | (2,46) | **0,006** |
| Cardiac Output | mL/min | 17,48 | (4,69) | 17,57 | (2,84) | 16,77 | (1,61) | 17,85 | (13,47) | 15,89 | (2,52) | 18,98 | (2,71) | 20,89 | (5,88) | 14,47 | (3,55) | **0,035** |

**Supplemental Table 2**

Echocardiographic parameters of WT and TLR3^-^/^-^mice at baseline and 2, 4, and 6 weeks following wire-injury of the aortic valve as illustrated in Figure 3, A.

|  |  | **Baseline** | | | | **Week 2** | | | | **Week 4** | | | | **Week 6** | | | |  |
| --- | --- | --- | --- | --- | --- | --- | --- | --- | --- | --- | --- | --- | --- | --- | --- | --- | --- | --- |
| Parameter | Value | WT | | TLR3 ^-^/^-^ | | WT | | TLR3 ^-^/^-^ | | WT | | TLR3 ^-^/^-^ | | WT | | TLR3 ^-^/^-^ | | P-value |
| **Suprasternal Axis** | | | | | | | | | | | | | | | | | | |
| VTI | mm | 46,35 | (6,28) | 46,21 | (6,19) | 72,36 | (17,72) | 61,16 | (8,40) | 74,19 | (19,33) | 64,85 | (15,03) | 69,51 | (21,45) | 54,97 | (11,70) | **0,029** |
| Mean Vel | mm/s | 549,83 | (66,41) | 547,77 | (61,58) | 878,24 | (179,45) | 731,96 | (113,48) | 910,59 | (224,62) | 791,23 | (116,68) | 890,10 | (260,23) | 681,42 | (113,29) | **0,008** |
| Mean Grad | mmHg | 1,23 | (0,30) | 1,21 | (0,28) | 3,21 | (1,22) | 2,19 | (0,66) | 3,51 | (1,65) | 2,56 | (0,75) | 3,42 | (2,19) | 1,91 | (0,61) | **0,016** |
| Peak Vel | mm/s | 1314,06 | (132,94) | 1246,24 | (177,64) | 2098,99 | (347,05) | 1680,77 | (319,89) | 2174,33 | (508,05) | 1797,14 | (292,36) | 2124,46 | (522,04) | 1636,84 | (326,40) | **0,005** |
| Peak Grad | mmHg | 6,97 | (1,43) | 6,33 | (1,87) | 18,07 | (6,19) | 11,68 | (4,73) | 19,87 | (8,58) | 13,24 | (4,25) | 19,07 | (9,78) | 11,11 | (4,58) | **0,008** |
| **Parasternal short axis** | | | | | | | | | | | | | | | | | | |
| Heart Rate | BPM | 414,03 | (38,06) | 425,38 | (34,08) | 441,46 | (44,83) | 436,52 | (42,38) | 434,24 | (36,20) | 474,65 | (58,98) | 426,23 | (33,68) | 466,50 | (49,46) | **0,013** |
| Diameter;s | mm | 3,05 | (0,42) | 3,29 | (0,31) | 3,07 | (0,30) | 3,26 | (0,29) | 3,32 | (0,39) | 3,08 | (0,38) | 3,14 | (0,36) | 3,19 | (0,31) | 0,687 |
| Diameter;d | mm | 4,07 | (0,35) | 4,17 | (0,33) | 4,15 | (0,27) | 4,21 | (0,28) | 4,29 | (0,34) | 4,02 | (0,37) | 4,10 | (0,29) | 4,02 | (0,26) | 0,433 |
| Volume;s | uL | 37,68 | (12,09) | 44,50 | (9,69) | 37,67 | (8,75) | 43,48 | (9,66) | 45,59 | (12,95) | 38,27 | (12,15) | 39,80 | (10,34) | 41,35 | (9,74) | 0,676 |
| Volume;d | uL | 73,57 | (14,76) | 77,75 | (13,83) | 76,92 | (11,78) | 79,72 | (12,47) | 83,32 | (15,63) | 71,50 | (16,09) | 74,73 | (12,00) | 71,46 | (10,99) | 0,443 |
| Stroke Volume | uL | 35,89 | (5,51) | 33,25 | (9,10) | 39,25 | (5,29) | 36,24 | (4,94) | 37,73 | (8,34) | 33,23 | (8,11) | 34,93 | (6,77) | 30,11 | (4,48) | 0,029 |
| Ejection Fraction | % | 49,82 | (8,31) | 42,48 | (10,52) | 51,48 | (5,75) | 45,82 | (4,71) | 45,75 | (8,66) | 46,86 | (8,52) | 47,24 | (8,79) | 42,63 | (6,64) | 0,116 |
| Fractional Shortening | % | 25,15 | (5,20) | 20,89 | (5,53) | 26,12 | (3,54) | 22,65 | (2,69) | 22,81 | (5,18) | 23,30 | (4,87) | 23,64 | (5,58) | 20,77 | (3,83) | 0,111 |
| Cardiac Output | mL/min | 14,86 | (2,68) | 14,17 | (4,07) | 17,23 | (2,24) | 15,87 | (2,98) | 16,35 | (3,74) | 15,68 | (3,84) | 14,91 | (3,28) | 14,02 | (2,36) | 0,401 |
| LV Mass | mg | 126,15 | (23,05) | 133,92 | (23,01) | 132,52 | (22,59) | 142,34 | (21,02) | 148,59 | (37,92) | 145,58 | (16,56) | 144,97 | (49,24) | 152,62 | (23,04) | 0,590 |
| LV Mass Cor | mg | 100,92 | (18,44) | 107,13 | (18,40) | 106,02 | (18,07) | 113,87 | (16,82) | 118,87 | (30,34) | 116,46 | (13,25) | 115,97 | (39,39) | 122,09 | (18,44) | 0,590 |
| LVAW;s | mm | 1,16 | (0,16) | 1,14 | (0,19) | 1,23 | (0,16) | 1,20 | (0,11) | 1,18 | (0,18) | 1,26 | (0,14) | 1,27 | (0,18) | 1,22 | (0,22) | 0,501 |
| LVAW;d | mm | 0,88 | (0,10) | 0,90 | (0,14) | 0,90 | (0,12) | 0,92 | (0,12) | 0,88 | (0,15) | 0,96 | (0,09) | 0,94 | (0,14) | 0,96 | (0,15) | 0,709 |
| LVPW;s | mm | 1,07 | (0,27) | 1,00 | (0,14) | 1,10 | (0,13) | 1,05 | (0,12) | 1,12 | (0,22) | 1,20 | (0,14) | 1,10 | (0,25) | 1,15 | (0,22) | 0,565 |
| LVPW;d | mm | 0,78 | (0,25) | 0,77 | (0,13) | 0,77 | (0,12) | 0,81 | (0,11) | 0,85 | (0,18) | 0,91 | (0,13) | 0,85 | (0,26) | 0,95 | (0,20) | 0,248 |
| **Parasternal long axis** | | | | | | | | | | | | | | | | | | |
| Heart Rate | BPM | 416,68 | (34,59) | 424,91 | (30,48) | 444,76 | (43,48) | 429,41 | (51,81) | 438,12 | (35,48) | 474,50 | (48,76) | 432,38 | (35,99) | 458,17 | (54,11) | 0,136 |
| Area | mm2 | 15,58 | (2,08) | 16,83 | (3,15) | 16,60 | (2,70) | 17,93 | (3,21) | 18,08 | (3,72) | 16,48 | (3,48) | 16,71 | (3,25) | 16,32 | (2,70) | 0,723 |
| Area;s | mm2 | 15,58 | (2,08) | 16,46 | (2,45) | 16,60 | (2,70) | 16,19 | (5,03) | 17,52 | (2,46) | 16,01 | (2,59) | 16,71 | (3,25) | 16,32 | (2,70) | 0,723 |
| Area;d | mm2 | 24,23 | (2,39) | 24,65 | (2,57) | 25,13 | (3,11) | 23,00 | (6,79) | 25,75 | (1,85) | 23,51 | (3,05) | 24,59 | (3,13) | 23,38 | (2,42) | 0,246 |
| Volume | uL | 30,81 | (6,92) | 36,11 | (11,60) | 33,27 | (7,94) | 39,73 | (12,98) | 39,12 | (14,37) | 34,52 | (13,39) | 34,52 | (11,61) | 33,13 | (7,91) | 0,704 |
| Volume;s | uL | 30,81 | (6,92) | 34,48 | (8,93) | 33,27 | (7,94) | 34,64 | (12,56) | 36,54 | (7,93) | 32,55 | (9,74) | 34,52 | (11,61) | 33,20 | (7,96) | 0,719 |
| Volume;d | uL | 64,68 | (10,83) | 68,37 | (11,21) | 69,64 | (13,60) | 63,27 | (20,54) | 69,38 | (7,98) | 62,27 | (13,58) | 66,50 | (13,87) | 61,41 | (10,94) | 0,274 |
| Stroke Volume | uL | 33,87 | (7,02) | 33,89 | (5,53) | 36,36 | (9,71) | 28,63 | (10,76) | 32,84 | (7,64) | 29,71 | (7,88) | 31,98 | (6,70) | 28,21 | (7,00) | 0,143 |
| Ejection Fraction | % | 52,36 | (6,87) | 50,10 | (7,38) | 52,06 | (8,25) | 42,32 | (14,20) | 47,39 | (9,83) | 48,01 | (8,51) | 48,97 | (9,56) | 46,07 | (8,83) | 0,395 |
| Fractional Shortening | % | 12,40 | (3,47) | 9,05 | (4,03) | 9,84 | (4,59) | 9,45 | (4,76) | 10,31 | (5,46) | 10,00 | (4,05) | 8,49 | (4,65) | 11,46 | (5,04) | 0,105 |
| Cardiac Output | mL/min | 14,12 | (3,27) | 14,44 | (2,81) | 15,94 | (3,71) | 12,36 | (5,21) | 14,47 | (3,94) | 13,99 | (3,35) | 13,85 | (3,06) | 12,86 | (3,18) | 0,392 |

**Supplemental Table 3:**

Echocardiographic parameters of WT mice treated with C4a or vehicle control (PBS) at baseline and 2, 4, and 6 weeks following wire-injury of the aortic valve as illustrated in Figure 5, C.

|  |  | **Baseline** | | | | **Week 2** | | | | **Week 4** | | | | **Week 6** | | | | p- value |
| --- | --- | --- | --- | --- | --- | --- | --- | --- | --- | --- | --- | --- | --- | --- | --- | --- | --- | --- |
| Parameter | Value | Vehicle | | C4a | | Vehicle | | C4a | | Vehicle | | C4a | | Vehicle | | C4a | |  |
| **Suprasternal Axis** | | | | | | | | | | | | | | | | | | |
| VTI | mm | 67,04 | (43,97) | 55,75 | (15,30) | 115,37 | (58,61) | 62,46 | (15,85) | 118,91 | (62,88) | 63,73 | (16,84) | 149,96 | (94,61) | 66,86 | (21,42) | **0,001** |
| Mean Vel | mm/s | 743,97 | (323,99) | 648,38 | (174,03) | 1004,92 | (253,22) | 685,16 | (187,35) | 1135,28 | (302,32) | 746,23 | (174,87) | 1137,89 | (448,28) | 680,87 | (169,30) | **0,001** |
| Mean Grad | mmHg | 2,61 | (3,37) | 1,80 | (1,04) | 4,28 | (2,13) | 2,01 | (1,08) | 5,50 | (2,86) | 2,34 | (1,10) | 5,93 | (5,29) | 1,96 | (0,97) | **0,005** |
| Peak Vel | mm/s | 1638,64 | (743,67) | 1472,36 | (327,76) | 2162,69 | (498,65) | 1595,47 | (284,01) | 2294,39 | (508,68) | 1594,82 | (286,18) | 2294,93 | (737,60) | 1510,39 | (279,04) | **0,001** |
| Peak Grad | mmHg | 12,83 | (17,33) | 9,08 | (4,62) | 19,65 | (9,34) | 10,49 | (3,58) | 22,03 | (9,44) | 10,48 | (3,72) | 23,11 | (15,99) | 9,42 | (3,40) | **0,002** |
| **Parasternal short axis** | | | | | | | | | | | | | | | | | | |
| Heart Rate | BPM | 400,90 | (25,40) | 408,53 | (39,62) | 460,85 | (44,39) | 420,92 | (37,61) | 469,74 | (39,04) | 448,07 | (44,35) | 475,54 | (34,08) | 456,62 | (36,27) | 0,133 |
| Diameter;s | mm | 2,70 | (0,36) | 2,63 | (0,31) | 2,90 | (0,24) | 3,08 | (0,21) | 3,02 | (0,29) | 3,04 | (0,28) | 2,89 | (0,36) | 3,19 | (0,34) | 0,02 |
| Diameter;d | mm | 3,75 | (0,33) | 3,73 | (0,28) | 3,84 | (0,15) | 3,94 | (0,14) | 3,94 | (0,27) | 3,94 | (0,28) | 3,97 | (0,25) | 4,06 | (0,30) | 0,358 |
| Volume;s | uL | 27,69 | (7,88) | 25,84 | (7,27) | 32,58 | (6,58) | 37,49 | (6,33) | 36,20 | (8,57) | 36,70 | (7,52) | 32,83 | (9,15) | 41,53 | (10,25) | **0,015** |
| Volume;d | uL | 60,75 | (11,66) | 59,89 | (10,36) | 63,72 | (6,01) | 67,60 | (5,61) | 67,92 | (10,00) | 68,00 | (11,14) | 69,02 | (10,09) | 73,29 | (12,24) | 0,284 |
| Stroke Volume | uL | 33,06 | (5,11) | 34,04 | (5,77) | 31,14 | (4,26) | 30,11 | (4,53) | 31,72 | (8,40) | 31,30 | (7,32) | 36,19 | (8,38) | 31,76 | (7,33) | 0,116 |
| Ejection Fraction | % | 55,34 | (7,00) | 57,34 | (7,08) | 49,15 | (7,14) | 44,67 | (6,80) | 46,51 | (10,22) | 45,96 | (8,22) | 52,71 | (10,46) | 43,67 | (8,56) | **0,010** |
| Fractional Shortening | % | 28,42 | (4,62) | 29,77 | (4,75) | 24,57 | (4,40) | 21,92 | (3,88) | 23,18 | (6,07) | 22,73 | (4,82) | 27,09 | (7,02) | 21,46 | (4,85) | **0,011** |
| Cardiac Output | mL/min | 13,29 | (2,41) | 13,98 | (2,99) | 14,42 | (2,86) | 12,73 | (2,42) | 15,00 | (4,52) | 13,99 | (3,29) | 17,40 | (5,03) | 14,51 | (3,58) | 0,065 |
| LV Mass | mg | 111,10 | (22,79) | 109,37 | (20,45) | 108,24 | (23,73) | 102,46 | (17,94) | 118,24 | (26,75) | 112,16 | (11,31) | 121,94 | (20,80) | 117,12 | (17,51) | 0,476 |
| LV Mass Cor | mg | 88,88 | (18,23) | 87,49 | (16,36) | 86,59 | (18,98) | 81,97 | (14,35) | 94,59 | (21,40) | 89,73 | (9,05) | 97,56 | (16,64) | 93,70 | (14,01) | 0,475 |
| LVAW;s | mm | 1,24 | (0,17) | 1,25 | (0,19) | 1,19 | (0,22) | 1,05 | (0,16) | 1,21 | (0,21) | 1,18 | (0,14) | 1,33 | (0,25) | 1,18 | (0,12) | 0,034 |
| LVAW;d | mm | 0,88 | (0,13) | 0,92 | (0,18) | 0,90 | (0,23) | 0,81 | (0,14) | 0,92 | (0,21) | 0,88 | (0,12) | 0,93 | (0,20) | 0,90 | (0,14) | 0,619 |
| LVPW;s | mm | 1,05 | (0,32) | 1,02 | (0,25) | 0,96 | (0,12) | 0,88 | (0,10) | 0,98 | (0,18) | 0,92 | (0,13) | 0,95 | (0,17) | 0,87 | (0,21) | 0,24 |
| LVPW;d | mm | 0,78 | (0,29) | 0,73 | (0,22) | 0,67 | (0,08) | 0,66 | (0,12) | 0,71 | (0,14) | 0,70 | (0,12) | 0,73 | (0,14) | 0,67 | (0,19) | 0,31 |
| **Parasternal long axis** | | | | | | | | | | | | | | | | | | |
| Heart Rate | BPM | 375,95 | (94,83) | 474,90 | (189,57) | 458,77 | (45,72) | 459,68 | (144,27) | 471,84 | (38,13) | 457,71 | (42,78) | 478,05 | (35,70) | 458,70 | (33,75) | 0,12 |
| Area | mm2 | 15,89 | (4,14) | 15,48 | (1,97) | 17,18 | (2,13) | 16,35 | (1,67) | 17,54 | (2,20) | 17,03 | (2,24) | 18,28 | (1,60) | 17,05 | (2,41) | 0,096 |
| Area;s | mm2 | 14,50 | (5,28) | 15,48 | (1,97) | 17,18 | (2,13) | 16,35 | (1,66) | 17,54 | (2,20) | 17,03 | (2,23) | 18,28 | (1,59) | 17,05 | (2,41) | 0,096 |
| Area;d | mm2 | 25,06 | (9,58) | 24,61 | (2,18) | 24,93 | (2,90) | 25,06 | (2,06) | 25,22 | (2,42) | 24,73 | (2,56) | 25,62 | (2,10) | 25,02 | (2,79) | 0,493 |
| Volume | uL | 33,56 | (10,68) | 29,48 | (5,43) | 35,69 | (7,00) | 33,13 | (5,35) | 37,22 | (8,45) | 35,30 | (7,71) | 39,94 | (5,91) | 35,87 | (7,86) | 0,104 |
| Volume;s | uL | 34,72 | (21,87) | 29,48 | (5,43) | 35,69 | (7,00) | 33,16 | (5,39) | 37,22 | (8,45) | 35,25 | (7,64) | 39,82 | (5,76) | 35,87 | (7,86) | 0,112 |
| Volume;d | uL | 63,43 | (18,37) | 65,32 | (8,98) | 67,97 | (13,27) | 67,51 | (7,47) | 69,89 | (11,25) | 66,68 | (10,56) | 71,03 | (8,15) | 69,41 | (11,57) | 0,647 |
| Stroke Volume | uL | 32,13 | (10,63) | 35,83 | (7,42) | 32,28 | (9,83) | 34,36 | (6,09) | 32,67 | (7,31) | 31,44 | (7,71) | 31,21 | (6,81) | 33,54 | (7,19) | 0,347 |
| Ejection Fraction | % | 49,64 | (16,33) | 54,68 | (7,10) | 47,00 | (8,26) | 50,75 | (6,57) | 46,83 | (7,71) | 47,09 | (8,46) | 43,71 | (7,03) | 48,36 | (7,33) | 0,073 |
| Fractional Shortening | % | 12,91 | (7,63) | 12,32 | (6,21) | 10,22 | (4,60) | 12,45 | (5,54) | 8,54 | (4,06) | 10,40 | (4,44) | 7,94 | (4,73) | 9,78 | (5,40) | 0,307 |
| Cardiac Output | mL/min | 12,68 | (4,77) | 16,89 | (6,92) | 14,79 | (4,67) | 15,87 | (6,32) | 15,33 | (3,29) | 14,24 | (3,16) | 14,90 | (3,45) | 15,43 | (3,68) | 0,673 |
